# A Causality-Inspired Approach for Anomaly Detection in a Water Treatment Testbed

**DOI:** 10.3390/s23010257

**Published:** 2022-12-27

**Authors:** Georgios Koutroulis, Belgin Mutlu, Roman Kern

**Affiliations:** 1 Pro2Future GmbH, 8010 Graz, Austria; 2 Institute of Interactive Systems and Data Science, Graz University of Technology, 8010 Graz, Austria; 3 Area of Knowledge Discovery, Know-Center GmbH, 8010 Graz, Austria

**Keywords:** anomaly detection, cyber-attacks, causal inference, convolutional neural networks, water treatment testbed

## Abstract

Critical infrastructure, such as water treatment facilities, largely relies on the effective functioning of industrial control systems (ICSs). Due to the wide adoption of high-speed network and digital infrastructure technologies, these systems are now highly interconnected not only to corporate networks but also to the public Internet, mostly for remote control and monitoring purposes. Sophisticated cyber-attacks may take advantage the increased interconnectedness or other security gaps of an ICS and infiltrate the system with devastating consequences to the economy, national security, and even human life. Due to the paramount importance of detecting and isolating these attacks, we propose an unsupervised anomaly detection approach that employs causal inference to construct a robust anomaly score in two phases. First, minimal domain knowledge via causal models helps identify critical interdependencies in the system, while univariate models contribute to individually learn the normal behavior of the system’s components. In the final phase, we employ the extreme studentized deviate (ESD) on the computed score to detect attacks and to exclude any irrelevant sensor signals. Our approach is validated on the widely used Secure Water Treatment (SWaT) benchmark, and it exhibits the highest F1 score with zero false alarms, which is extremely important for real-world deployment.

## 1. Introduction

Modern urban development along with the advent of the digitalization era has inevitably led to the wide adoption of cyber–physical systems (CPS) in critical infrastructure, such as power generation and distribution, health care facilities, water treatment plants, and more [1]. Principally, a CPS consists of three components: the physical process, the computational layer, and the digital network infrastructure that ensures a two-way communication between the former components. In addition to their architectural complexity with multiple heterogeneous components, CPSs are usually controlled remotely either via public or private networks, which renders them extremely vulnerable to cyber-attacks. In 2015 alone, the US Department of Homeland Security (DHS) reported 25 cyber-attacks in the water sector and 46 in the energy sector [2]. More recently, in 2021, attackers successfully infiltrated a water treatment facility of the City of Oldsmar in Florida to make the water unsafe for consumption by manipulating specific chemical levels [3]. Previous security incidents in water systems, as well as many others [4], highlight the importance of developing solutions that can effectively detect cyber-attacks and eliminate their impact on the normal operation of the entire CPS.

**Figure 1:**
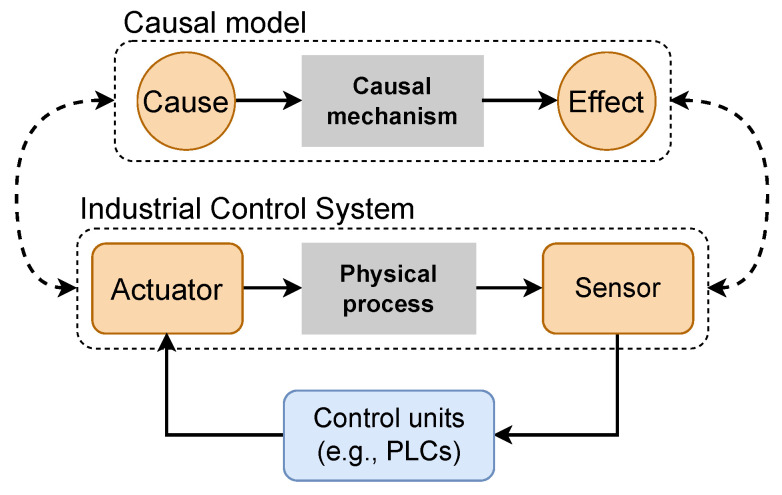
Analogy between the main components of a typical ICS and a causality-based model.

Among the several types of CPSs, an Industrial Control System (ICS) typically integrates interconnected devices for monitoring and controlling a physical process in critical infrastructure. Specifically, multiple sensors record physical quantities (e.g., pressure and tank level) that are processed by high-level computing elements such as Programmable Logical Controllers (PLC). After processing the signals from the sensors and subsequently computing the corresponding actions via a prescribed control logic, PLCs send action signals to actuators (e.g., valves and pumps), ensuring all design specifications are met. We use the following example to show the physical connection between an actuator and a sensor within an ICS. In a water treatment plant, moving the diaphragm of a valve to a specific position increases or decreases the flow into a downstream tank, which is governed by a physical law. Inspired by the notion of causality, we illustrate in Figure 1 the analogy between the connection of the physical components in an ICS with the causal model that represents a mathematical abstraction between the cause and the effect. Based on this principle, we aim to propose a causal-based approach for effectively detecting attacks in an ICS.

Cyber attacks are usually targeted at critical components of an ICS for the purpose of disrupting the associated physical process and ultimately causing plant damage with major consequences. The attacks are reflected in the data as anomalies as soon as the system’s operation diverges from its design bounds and enters the unexpected abnormal state. Since all types of abnormal states may not be available beforehand, data-driven methods that incorporate unsupervised learning are often preferred over supervised ones [5]. Another reason is the dominance of normal operating conditions in an ICS, while anomalies due to deliberate attacks or other software-hardware failures rarely occur. However, unsupervised learning techniques are prone to detecting false alarms due to highly complex and noisy data. It is important to keep in mind that an attack detection system should generate at most one false alarm in six months [5]; otherwise, it might be impractical for real-world deployment. To this end, novel unsupervised techniques with highly denoising properties are needed that can eliminate any false alarm without sacrificing the overall detection rate of unknown attacks.

Unsupervised deep learning techniques have already demonstrated promising results to detect attacks in complex ICSs [6,7,8,9]. In particular, convolutional neural network (CNN) architectures are able to automatically extract meaningful features from time series and learn unique temporal patterns under normal operational conditions. Any unexpected deviation in the observations that is present due to cyber-attacks is captured by the model’s prediction errors. In a similar context, Long Short-Term Memory neural networks (LSTM) [6] are able to model very long-term temporal dependencies in sequential data, which in practice can be quite computationally intensive due to their high number of learning parameters. In general, a major drawback of such deep learning architectures is that they solely learn from correlations in the data. Therefore, causal structures that can render the model more robust under distribution shifts and noisy environments are likely to be overlooked [10,11,12]. Another inherent issue is the lack of interpretability of black-box models [13] since it is infeasible to understand the prediction mechanism for specific outputs. Finally, incorporating the time series of all sensor measurements and actuator states might lead to increased training times and degradation of the model’s performance due to overfitting.

Univariate models [14,15] can partly alleviate the curse of dimensionality issue since autoregression forecasting models can be generated individually from each transmitted signal within an ICS. Their implementation is relatively easy, and they can be locally deployed directly in control system devices so that latency issues are avoided. Unfortunately, in the univariate case, no temporal dependencies among the sensors can be captured, let alone any latent causality in the data. Nevertheless, univariate models could point out possible root-causes of the detected attacks if designed carefully with respect to eliminating false alarms and detecting the sensor measurements that are causally connected with the attacked sensor/actuator. Despite the high computational cost that may arise by the implementation of multivariate detection models, incorporating only the necessary design knowledge to build these models can mitigate the above challenges and generate robust solutions [16,17]. To this end, we aim to include both univariate and multivariate models to obtain state-of-the-art performance.

To address the aforementioned challenges, we propose a causality-inspired unsupervised learning approach for detecting cyber-attacks in water treatment plants. Our framework consists of two main stages, namely an offline and an online phase. In the offline phase, multivariate models are constructed from structural causal models [18] that entail the underlying physical mechanism of the corresponding process stages. A minimum amount of domain knowledge is needed to define these structures. Furthermore, univariate models are built from continuous sensor signals that may indicate not only the effects of the attacks but potential root causes. In the online phase, a denoised anomaly score is computed by a causal inference model, and the final decision of an attack is inferred via a hypothesis test. In case a univariate model does not contribute to the final attack detection, it is removed from the final set of the true root cause. Ultimately, the final set of parameters results in finding the root cause candidates. Encoding our assumptions in a causal graph enables us to isolate the noise from external factors and to estimate with transparency an unbiased estimate of the causal effect of an attack to the prediction errors that are derived from the previous models. Finally, our method is evaluated with data from the Secure Water Treatment (SWaT) testbed, which was originally built at the Singapore University of Technology and Design for cybersecurity research [19].

The main contributions of this work are summarized as follows:

1. A novel anomaly score based on causal inference is proposed and is computed with a linear regression approach. Low model complexity due to linear computation renders it highly appropriate for demanding streaming environments.
2. Our detector not only achieves a high detection rate, but at the same time also highlights the severity levels of the attacked components by incorporating recovery periods. By denoising the prediction errors with the causal effect estimation, we achieve a zero false alarm rate on the test data.
3. Unlike most of the previous works [20], in our approach, no predefined threshold is needed to decide if an attack is present or not since the extreme stundentized deviate (ESD) [21] is applied on the denoised errors. To the best of our knowledge, there is no previous work for attack detection in ICSs that utilize the ESD test due to its normality constraint in the raw data.
4. A selection technique for indicating the root cause candidates from the univariate prediction models is performed in the online phase, which yields the set of sensor signals that are largely associated with the launched attacks. Providing the potential root causes of an anomaly will further help process operators to choose appropriate mitigation actions.

Section 2 summarizes related work and discusses limitations in each of the methods. Section 3 introduces the proposed approach with the necessary theoretical background. Section 4 describes a case study using historical data from the SWaT testbed and compares the performance of the proposed approach with other methods. Section 5 summarizes the results of our analyses and potential future work to augment or improve the proposed approach.

## 2. Related Work

A growing body of literature in cyber-security for critical infrastructure [22,23,24,25] has already been devoted to detecting and preventing cyber-attack infiltration. Recently, Tuptuk et al. [26] systematically reviewed and categorized state-of-the-art methodologies on cyber-security in water infrastructure that largely integrate smart technologies. According to the research conducted in the review, the investigation of detection solutions in CPS gained the most attention from the scientific community over the last 15 years, thus highlighting the magnitude of this problem. In this section, we present related work that focuses on detection of cyber attack techniques for water systems that mostly employ unsupervised machine learning methods. However, additional works on process invariants are presented that conceptually resemble our causality-based approach to some extent.

Besides applications in CPS, anomaly detection techniques have been widely devised within other research areas as well. Specifically, in the information technology sector, Djurasevic et al. [27] presented a failure detection method for hard disc drives that utilizes a specific set of monitoring and diagnostic parameters to train a support vector machine classifier. A recursive feature elimination approach is further employed to select the most important features from the overall set that contribute the most to the detection of the anomalies. In the field of forensic handwriting verification, Mazzolini et al. [28] proposed a framework for detecting imitated digital signatures from dynamic features, and the authors used the distributional changes of these features to enhance the interpretability for the final decision of an non-genuine signature. Finally, in [29], Corizzo et al. proposed an anomaly detection framework that encodes the geographical location of renewable energy plants with multivariate sensor data. A distance-based metric was computed, and based on a manually defined threshold, anomalies were finally predicted. Due to the unsupervised learning scheme and the spatial encoding deep learning architecture, the method yielded very high precision and outperformed all other competitors. As we show next, unsupervised techniques might be the best candidates for real-world anomaly detection applications.

Due to the lack of annotated anomalies in practice, unsupervised learning has already been widely established for anomaly and attack detection purposes in CPSs [23,30,31]. Goh et al. [6] first proposed an unsupervised learning technique for attack detection in a water treatment plant by employing and stacking together multiple layers of Long Short-Term Memory (LSTM) networks that solve the vanishing/exploding gradient issue from traditional recurrent neural networks (RNN). A cumulative sum (CUSUM) method is then adopted for constructing an anomaly score that may address high false positive rates due to threshold sensitivities. However, a manually predefined threshold is necessary, thus rendering CUSUM less robust for dynamical operating conditions. Additionally, the authors also reported insufficient evaluation of the method, since it was only validated in the first process stage of the SWaT dataset. Similarly, Raman et al. [15] trained a Multi-Layer Perceptron (MLP) neural network model to learn the normal physical behavior of the system and subsequently computed CUSUM to identify the injected attacks. The authors evaluated the MLP-based method with attacks that were directly influencing process P1 of the SWaT testbed, and hence only measured parameters from this stage were used. Inoue et al. [7] proposed a deep neural network architecture (DNN) with LSTM layers that accounts for both sensor and actuator data from all process stages. The DNN method is compared with a window-based one-class Support Vector Machine (SVM), and the final evaluation showed that DNN is slightly better than SVM with regards to fewer false alarms. Building upon the previous works, Kravchik and Shabtai [32] built distributed deep learning models for each process stage with 1D convolutional neural networks (1D-CNN ensemble) that outperformed the state-of-the-art. The authors also employed a single combined model with CNN for all stages of the SWaT that still outperformed all other methods except the 1D-CNN ensemble approach, thus highlighting the power of CNN in attack detection. Our unsupervised approach similarly adopts a distributed concept of the causal models, since a causal mechanism in an individual phase is a data-based deterministic representation of the underlying physics.

Despite the high performance of deep learning architectures in attack detection scenarios, they generally suffer from excessive hyperparameter tuning costs since numerous parameters of the predictive models, such as learning rate, number of epochs, type of activation function, etc., need to be appropriately selected [20]. To address this challenge, the authors in [9] proposed an LSTM variational autoencoder neural network, and they claimed to have achieved rather comparable performance via a lightweight model architecture. Further, a feature selection technique is performed on the sensor/actuator signals from the SWaT based on the Kolmogorov–Smirnov test (K–S test) in a supervised manner, which might have some limitations in real-world conditions. Instead, our selection approach for the root cause candidates is performed based on the causality-based anomaly score without any prior information of the injected attack scenario. Kravchik and Shabtai [33] likewise proposed a lightweight neural network architecture for attack detection scenarios by employing 1D CNN and autoencoders. Although most significant hyperparameters were selected based on grid search and genetic algorithms, the final threshold needs to be set manually, which can be considered a drawback in practical scenarios. Shalyga et al. [34] applied genetic algorithms to optimize the hyperparameters from an encoder–analyzer–decoder architecture based on three types of neural networks, namely, multilayer perceptron, convolutional networks, and recurrent neural network. Surprisingly, the optimized MLP architecture achieved the highest performance on the SWaT testbed in terms of F1 score. Recently, Nedeljkovic and Jakovljevic in [14] developed a method that consisted of several one-step forecasting univariate 1D-CNN models that are trained with attack-free data. Unlike our approach, only sensor signals are used, and the hyperparameters of the CNN architectures are individually optimized with a grid-based strategy for each signal separately.

Previous unsupervised approaches mostly utilize machine learning models to learn the normal behavior from raw time series and compute an anomaly score based on the prediction errors in the test data. However, clustering-based methods use a rather different approach to detect attacks in ICS. The authors in [35] proposed CD-OCC, a student–teacher learning strategy that combines a K-Means clustering algorithm with deep learning (DL) models. K-Means was only trained with the normal operation set, and later the derived cluster labels were used to obtain softmax values by training the DL classifier models. Further, a one-class classification algorithm (e.g., isolation forest [36]) was employed to detect the presence of an anomaly. A final evaluation was performed on the SWaT testbed and showed that deep neural networks with isolation forest outperformed all other methods. Elnour et al. [37] proposed a hybrid method for attack detection on the SWaT testbed that initially employs a 1D CNN for automatically extracting meaningful features from the continuous sensor signals, which later are combined with the discrete actuator signals to train the isolation forest model. In a later work from Elnour et al. [38], the authors developed a method based on dual isolation forests (DIF), in which one model is trained with the raw actuator/sensor data and the other one with data from a low-dimensional space using principal component analysis (PCA). Both previous studies [37,38] highlight the importance of accounting for both types of signals on the final attack detection performance, which our method also embraces.

Differentiating substantially from purely data-driven approaches, invariant-based methods utilize the conditions that must hold, whenever the system is in a given state according to its design specifications [39]. Usually, design knowledge is required for extracting such process invariants. For example, recently AICrit [17] utilized the domain knowledge obtained by the Process and Instrumentation Diagram (P & ID) to construct ten invariants than contain both sensor and actuator parameters from the SWaT testbed. A zero false positive rate is accomplished by the model; however, only 24 out of 36 attack scenarios from the evaluation on the SWaT dataset are detected. It should be noted that the method did not detect any stealthy attacks due to their low manipulation rate on the sensor values. A drawback of invariant-based methods is that in very complex ICSs, it may be quite cumbersome to manually include all possible invariants that yield the underlying physical or chemical properties of the system in multiple modes of operation. Hence, specific attacks might be missed from the corresponding detection models. To this end, Yoong and Heng [40] developed an invariant search mechanism by including grouping and adjacency constraints for constructing the final set of machine learning (ML) invariants. Autoregressive models with exogenous input variables (ARX) are employed to infer these ML invariants that are evaluated on the SWaT dataset. Resulting invariants on the SWaT testbed from the previous method also highlight the applicability and the potential of incorporating domain knowledge into data-driven techniques.

In contrast to previous works, causality-based methods have gained rather less attention in the large body of research in anomaly detection within ICSs. In general, causality analysis is divided into two main tasks. First, identifying the true causal structure from observation data falls into the scope of *causal discovery* [41,42]. Second, the task of *causal inference* [18,43] focuses on estimating causal effects from observational data between a treatment and an outcome variable provided that the overall causal structure is known. Although there are a few works that implement the former methods into their frameworks, as we show here, we identified a research gap in the literature since we did not find any work that includes causal inference techniques for anomaly detection in water systems. Regarding the first causal paradigm, Lin et al. [44] proposed a framework that first employs timed automata to model the normal sensor dynamics and Bayesian networks to learn the causal dependencies between sensors and actuators from the SWaT testbed. Due to the graphical nature of the method, it is highly interpretable and may provide additional insight to experts about the localization of the detected anomalies. ICS-CAD [45] is unfolded in two analysis phases. In the first phase, a deep learning sequence classification model is prepared for detecting attacks. When an attack is detected, in the second phase, a causal decomposition is performed to assess the impact of the attack on other system devices. An important limitation of the method is that labeled data of the anomalies must be available to train the supervised classifier. Furthermore, the performance of the causal decomposition method is significantly affected when detected attacks largely contain constant values, as it is in SWaT. Most recently, Yang et al. [46] employed causal discovery algorithms to automatically infer individual causal mechanisms (causal graphs), which are used to construct multivariate prediction models. By this way, complex anomalies can be better captured and isolated due to their local mechanisms. Finally, it should be noted that we do not use any causal discovery algorithm to infer the relations for the multivariate models, as such algorithms may be computationally expensive for large non-linear datasets [47] and hence less applicable for online deployment. Instead, we utilize the existing domain knowledge to include only the minimum necessary causal mechanisms.

## 3. Proposed Approach

### 3.1. Preliminary: Causal Inference

In this section, we introduce the fundamentals of causal inference that our approach employs for constructing the anomaly score. A basic notation is presented below, which is later transferred for estimating the causal effect in the temporal domain.

Let (X, c, e) be a standard dataset in which we want to estimate the causal effect of a binary treatment variable (cause) c ∈ {0,1} on a continuous outcome variable (effect) e ∈ R. Further, we assume a feature matrix vector X that contains all *confounders*, namely the variables that are causally influencing both the treatment and the outcome. Note that all confounders are observed, otherwise special causal effect learning methods need to be deployed [43,48]. Formally, the causal relations are represented graphically with Directed Acyclic Graphs (DAG), which means that the arrangement and orientation of the edges should not form any cycles.

**Figure 2:**
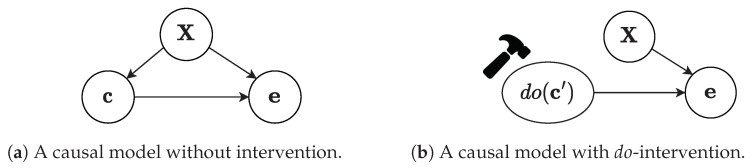
Our causal model with and without intervention.

Figure 2a shows the DAG that is entailed by the above causal model and also happens to be the focus of our study. However, the graph with this specific formation cannot account for any interventions on the treatment variable, and hence it is not possible to obtain an unbiased estimate of the causal effect on the outcome. For that reason, do-operator, denoted as $do(c\;=\;c^{\prime })$, is introduced by Pearl [18] and represents active manipulation of the graph, depicted in Figure 2b, by deleting all incoming edges on the treatment and setting the value of the variable *c* to $c^{\prime }$. The modified causal graph enables us to formulate the interventional distribution $P(e|do\left(c^{\prime }\right))$, an essential component for making quantitative predictions on the effects of interventions.

In supervised learning scenarios with continuous targets, such as regression, a linear/non-linear function is fitted to the data to obtain the conditional (observational) distribution P(y|X), where X are the input features and *y* is the target variable. In causality, similarly, a single function is fitted to the data from the intervened graph to estimate the interventional distribution P(e|do(c),X). This estimator serves as the mean to address the main challenge of causal inference, namely the observation of only one state (with or without treatment) for an individual instance in the data. For example, given an instance *i* that takes a treatment $c_{i}\;=\;1$, only the outcome $e_{i}^{1}$ is observed, while the outcome without any treatment $e_{i}^{0}$ is inferred by the regression model based on the features x and the treatment *c*. This procedure continues until the missing outcome values for all instances are imputed accordingly. Hence, over the entire dataset with i = 1, ⋯, N, the *average treatment effect* (ATE) can be formulated as follows:

(1) $$ATE\;=\;E\left[e|do\left(c\;=\;1\right)\right]\;-\;E\left[e|do\left(c\;=\;0\right)\right]\;=\frac{1}{N}\sum_{i=1}^{N}\left(e_{i}^{1}\;-\;e_{i}^{0}\right)$$

Since we defined the introductory theory of causal inference, we present below *structural causal models* (SCM)—the fundamental cornerstone that we use for building multivariate prediction models. Assuming a causal model $C_{j}\;\to \;E,\;j\;\in \;\left\{1,\;2,\;\cdots ,\;m\right\}$ with *m* multiple causes, it can be expressed by a structural equation as follows:

(2) $$E\;:=f(C_{j},\;\epsilon )$$

where *f* a deterministic function that entails the data generating mechanism, and ϵ is an independent noise factor. In our attack detection scenarios, we set effect variables as sensors, which are, in practice, influenced physically by individual actuators in terms of causal interventions. We consider the required domain knowledge as sufficiently low for building the SCMs in most of the practical applications.

### 3.2. Preliminary: Convolution Neural Networks

Convolutional Neural Networks (CNNs), originally developed for computer vision, have already shown exceptional performance for a wider range of time-series-related tasks, such as classification [49] and forecasting [50]. Typically, a CNN consists of stacked arrangements of convolutional, pooling and fully-connected layers. By sweeping the input temporal data with 1D convolutions of specifically sized kernels, shared weights in each layer are learned via a back-propagation algorithm. These weights are used to generate feature maps with specific dimension sizes that are likewise convolved and processed in later layers. Intuitively, feature maps incorporate either local patterns or dependencies, which in other cases would be extremely tedious to trace within very large time-series data. On the other hand, via pooling layers, down-sampling (e.g., averaging aggregation) from upstream feature maps is performed, without any actual parameter learning, to increase model’s robustness by reducing the sensitivity of the output to temporal shifts and distortions.

In this paper, the input data are either univariate and contain only one feature or are multivariate and depend on the selected feature set. For simplicity, we present the following notation with univariate input format. Let $X_{t}$, t ∈ Z be the input temporal sequence, and our goal is to build a one-step-ahead forecasting model based on the CNN architecture. Since we need to model temporal dependencies between current and past time points, a sliding window approach is applied on the original sequence. Hence, an individual window with a fixed length *l* is defined as:

(3) $$W_{t-l:t-1}=\left(x_{t-l},\;\ldots ,\;x_{t-2},\;x_{t-1}\right).$$

The output $h^{k+1}$ of an intermediate convolutional layer k+1 is obtained by applying a convolution operation over the input windowed data, or generally, over the output from the layer *k*:

(4) $$h^{k+1}=g\left(w^{k}*h^{k}\right)$$

where $w^{k}$ is a local kernel with a fixed size at layer *k*, ∗ denotes the convolution operator, and g(·) is an activation function that introduces non-linearity to the data.

**Figure 3:**
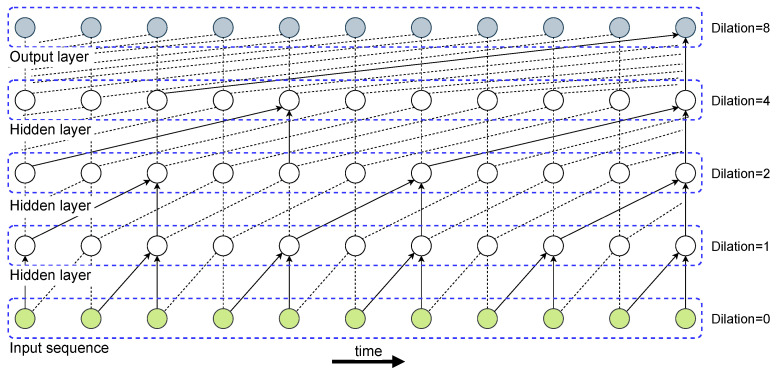
Representation of a stack of causal convolutional layers with different dilation sizes.

In this work, we extend the functionality of traditional 1D convolutional architectures by employing causal convolutional layers with dilations [51]. Inspired by causality’s notion that causal dependencies should coincide with the direction of time, the former convolutions apply causal filters for which their inputs can be only connected to future time steps in order to maintain the temporal ordering during training. Although causal convolutions are computationally easier to handle than RNNs, they require many layers to increase the area of the input sequence that affects a connected area in the output layer. Another issue is that very large kernel sizes are required to capture long-range dependencies, which might greatly inflate the computational cost. To address the previous shortcomings, dilated convolutions are introduced and have already shown outstanding performance for time-series forecasting problems [52]. Figure 3 illustrates dilated casual convolutions with dilation sizes 1, 2, 4, and 8 in a stacked manner that enables better generalization capabilities.

### 3.3. Preliminary: Extreme Studentized Deviate (ESD)

Extreme Studentized Deviate (ESD) [21] is a statistical technique for detecting multiple outliers in univariate sequence data that approximately follows a normal distribution. Let us now define an input sequence $X\;=\;\{x_{1},\;x_{2},\;\cdots ,\;x_{n}\}$ and *u* an upper bound of the number of the outliers. ESD performs a hypothesis test with the null hypothesis ($H_{0}$) that no outliers exist in the data, and the alternative hypothesis ($H_{a}$) is that up to *u* outliers are present in the data. The following test statistic is defined:

(5) $$C_{i}\;=\frac{\max_{i}\left|x_{i}\;-\;\bar{x}\right|}{s}$$

where i ∈ {1, 2, ⋯, u}, and $\bar{x}$ and *s* denote the mean and the standard deviation, respectively. Since our approach evaluates online data, critical values are compared with the test statistic up to the current point to determine whether the value is anomalous. If the value is an outlier, it is removed, and test statistic values are recalculated with the remaining observations. The corresponding critical values are computed by:

(6) $$\lambda_{i}\;=\;\frac{\left(n\;-\;i\right)t_{p,n-i-1}}{\sqrt{\left(n\;-\;i\;-\;1\;+\;t_{p,n-i-1}^{2}\right)\left(n\;-\;i\;+\;1\right)}}$$

(7) $$p\;=\;1\;-\frac{\alpha }{2(n\;-\;i\;+\;1)}$$

where α is the significance level, and $t_{p,v}$ is the 100*p* percentage point from the *t*-distribution with *v* degrees of freedom. ESD can repeats up to $u^{\prime }$ times ($u^{\prime }\;<\;u$), depending of the number of the anomalies, in which the condition $C_{i}\;>\;\lambda_{i}$ holds.

### 3.4. Causality-Inspired Anomaly Detection

**Figure 4:**
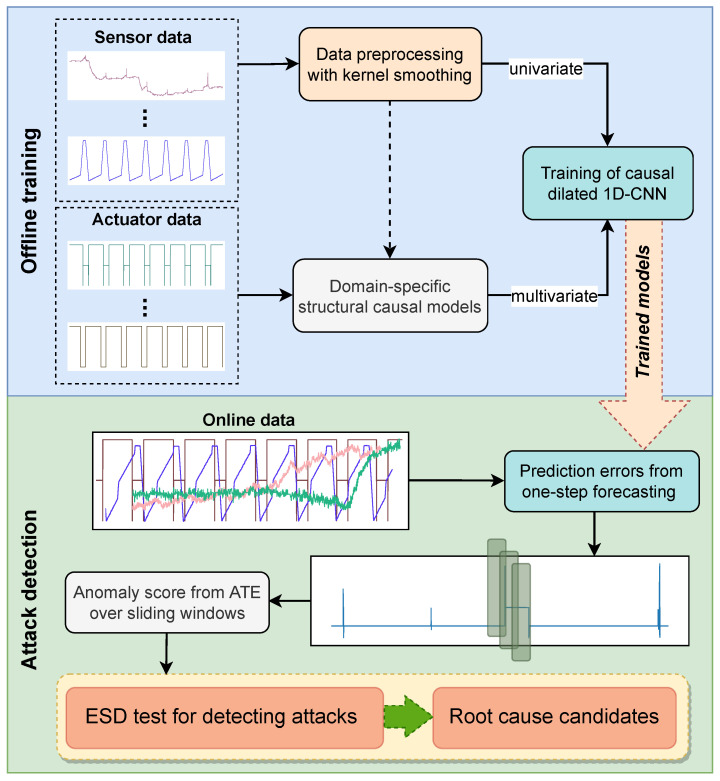
Overview of the proposed causality-inspired approach with offline and online phases. In the offline phase, training based on CNN forecasting generates both univariate and multivariate models. New data from sensor measurements and actuator states are used in the online phase to compute the ATE from the prediction errors over sliding windows. ESD test is finally performed on the aggregated sequence of the causal effect, first to detect anomaly status from incoming attacks, and second to indicate a set of the root cause candidates.

The proposed anomaly detecting approach consists of two main phases: (1) establishing univariate/multivariate prediction models via offline training with attack-free data, and (2) online attack detection with selection of the root cause candidates. Figure 4 illustrates a general overview with both phases and all the intermediate steps. While building univariate models is more straightforward and trivial, in a multivariate case this might not hold. We utilize domain knowledge to first select the physical “manipulators” (actuators) from the earliest process stages with all the sensors in the immediate vicinity. These interdependencies can be represented mathematically by structural causal models that are essentially used to construct the multivariate forecasting models. When all models are trained, prediction errors can be computed from online data that may contain various attack scenarios. On each error sequence, a sliding window is applied to compute the average treatment effect based on linear regression since we assume that our treatment variable (attack/no attack) is binary and the outcome (error) contains real values. Low model complexity of the method enables fast evaluation times, which is also appropriate for streaming environments. Finally, a hypothesis test is applied on the aggregated causal effect sequences to detect the existence of anomalous windows, while final results are also beneficial for creating sparsity for selecting the right candidate parameters.

#### 3.4.1. Data Pre-Processing

**Figure 5:**
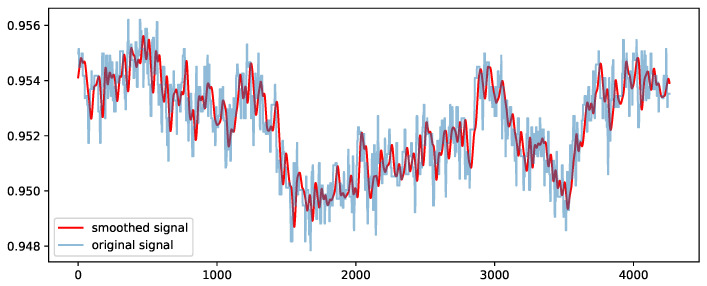
Application of the kernel smoothing technique on the normalized values of a sensor signal from the SWaT dataset.

In the offline phase, we carry out a pre-processing step of the dataset. In particular, raw time series of each sensor values are normalized with a min–max scaler for squashing the values into the range of [0, 1]. This is mainly performed to avoid deterioration of the performance during training of the CNN models. In addition to min–max normalization, we likewise apply a kernel smoothing technique [53] on the sensor (continuous) values with a Gaussian kernel and a bandwidth of 40 samples that defines the spread of the kernel. A moderate size value of the bandwidth is selected since a very small size might incorporate high-frequency noise and a large one might eliminate essential patterns in the time series. Figure 5 presents the results of the kernel-smoothing method using an example of a sensor’s signal values from the SWaT dataset.

#### 3.4.2. Neural Model Architecture

**Figure 6:**
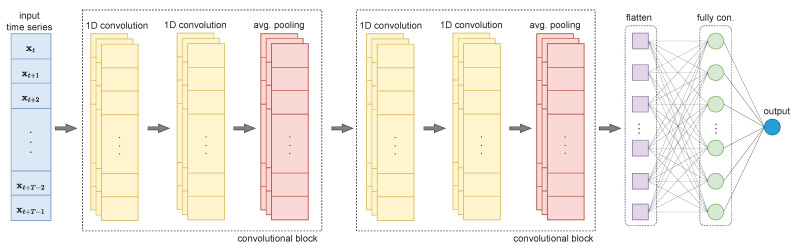
The 1D convolutional neural network architecture with two convolutional blocks. Depending on the anomaly detector type, input time series are either univariate or multivariate.

After definition of both families of models and data pre-processing, we introduce a lightweight deep learning architecture. A single 1D CNN architecture with causal dilations is developed for both univariate and multivariate models to avoid high computational costs from multiple hyperparameter tuning [20]. Within our research, we use the general architecture that is depicted in Figure 6 with all its building blocks. The architecture starts with two convolutional blocks, in which each contains two 1D convolutional layers that are followed by average pooling. Average pooling basically achieves a significant reduction in the computational cost since fewer parameters are learned, and hence, may lead to better generalization. The output of the last average pooling is the input of the flattening layer that, accordingly, adjusts the shape of the multi-dimensional data structures. Finally, a fully connected layer with a single output value yields a single-step forecasting of the selected sensor measurements. In the experiments with the SwaT dataset, we proceed with tuning all critical hyperparameters from the corresponding architecture.

#### 3.4.3. Anomaly Score

The majority of the existing methods in anomaly detection for CPS utilize either prediction or reconstruction error values [20]. In the former type of error, past data from sensor and actuator values are used as input to predict future sensor values, since target values are continuous. In the latter case, autoencoder models are usually employed to estimate the reconstruction error. In the proposed approach, we resort to *prediction errors* due to the forecasting scheme and apply causal inference techniques to denoise them.

For the sake of simplicity, we first formulate the anomaly detection problem for univariate models as follows. First, let $X_{t}^{j}$ denote a univariate time series for sensor *j*, and F(·) represents the causal dilated 1D-CNN model of the corresponding sensor variable, in which a non-linear mapping of the input window sequence $W_{t-l:t-1}^{j}$ to the one-step-ahead value $X_{t}^{j}$ is learned, namely $F\left(W_{t-l:t-1}^{j}\right)\;={\hat{X}}_{t}^{j}$. We then obtain predictions ${\hat{X}}_{t}^{j}$, and the error values at time *t* are defined as follows:

(8) $$e_{t}^{j}\;=\;\left|{\hat{X}}_{t}^{j}\;-\;X_{t}^{j}\right|,$$

where $X_{t}^{j}$ are the actual values from sensor *j*. Errors are estimated in both $D^{train}$ and $D^{w}$, which represent training (attack-free) and testing sliding windows (possible attacks) of sizes *N* and *m*, respectively. Here, we sample the previous error sequences using a sliding window with a fixed length *m*, where m >> l. A binary vector of values {0, 1} with size N + m, denoted by $c\;=\;\{c^{0},\;c^{1}\}$, is given by the concatenation of a treatment vector $c^{0}$ with zeros, where “0” means no attack is present; and a vector $c^{1}$ with ones, where “1” means an attack may be present. In a similar way, and for the corresponding samples, an effect vector $e\;=\;\{e^{0},\;e^{1}\}$ is obtained by the previous prediction errors. Finally, we generate the dataset ${\left\{X_{j},\;c,\;e\right\}}_{0}^{N+m}$ for each sliding window to estimate the causal effect of the treatment (attack/no attack) on the effect (prediction errors).

## 4. Case Study

The performance of the causal-based method is evaluated with the widely used Secure Water Treatment testbed (SWaT) [54]. In the following section, we provide a full overview with all the details regarding normal operation and operation under different attack scenarios. During the offline step, univariate and multivariate one-step forecasting models are trained with the proposed CNN-based architecture. We used normal behavior data from the SWaT dataset that subsequently were divided into 80% for training and 20% for tuning the hyperparameters of the proposed CNN architecture. A grid-search strategy resulted in the hyperparameter values that are presented in Table 1. Although the final hyperparameter values were slightly different for each model, we decided to keep these values as they yielded the lowest model complexity. The values listed below refer to both univariate and multivariate models, with the only difference being the number of neurons in the last fully connected layer.

Since one of the main goals of our proposed approach is to build a compact and fast method for online detection of cyber-attacks in ICSs, we opted to develop a computational architecture that can easily work with laptop-class processing power. Hence, the training of all deep learning models was performed using Google’s TensorFlow framework version 2.8.0 on an 8-core CPU MacBook Pro laptop equipped with an 8-core GPU Apple M1 chip and 16 GB RAM. It is worth noting that no GPU resources are utilized since our light CNN architecture can be easily trained solely on the CPU. CNN models were trained using Adam optimizer with a mean squared error loss function and learning rate of 0.001. All convolutional layers used the rectified linear unit (ReLU) as an activation function. Finally, a batch size of 1024 was used, and this resulted in a runtime per epoch of 20 s. Overall, the average training time was approximately 300 s, and training curves showed that validation loss reaches a low plateau after 20 epochs, which confirms our choice of the aforementioned hyperparameters.

### 4.1. SWaT Dataset

**Figure 7:**
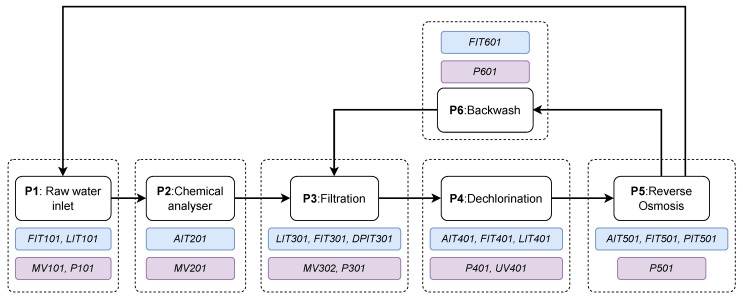
Process stages in the SWaT water treatment plant. Components that are enclosed in blue rectangles denote sensors, while actuators are enclosed in purple rectangles.

In our case study, we used the SWaT testbed to compare and evaluate the proposed approach. The SWaT testbed is a scaled down version of an ICS from a fully operational water treatment plant with all required software and hardware components that is capable of producing 5 gallons per minute of filtered water. Figure 7 provides a schematic illustration of the SWaT process divided into six stages, denoted P1 to P6. At the beginning of the treatment stage, P1, raw water’s flow is controlled via a motorized valve MV101, and it is stored in a tank in which its level is monitored by sensor LIT101. The valve receives the level signal to control the inflow via a PLC since the water level in the tank must be within a low and a high threshold value. Similarly, a downstream pump P101 turns on when the level drops, and accordingly, turns off when the level rises. Next, pre-treatment of the raw water is performed in the chemical dosing stage (P2) depending on the contaminant levels measured from the water-quality sensors. However, any undesirable solids are further filtered out in the ultrafiltration stage (P3). Dechlorination is performed in Stage 4 (P4) via ultraviolet lamps to remove any excessive free chlorine in the water. In Stage 5 (P5), the dechlorinated water is passed through a two-phase reverse osmosis filtration unit to further remove any inorganic impurities. Finally, Stage 6 (P6) ensures that a backwash process takes place to further clean the residuals from the ultrafiltration, while the filtered water is recycled into Stage 1.

The dataset from the SWaT testbed mainly consists of two subsets that contain a total of eleven days of recording from sensory and actuation data with a sampling rate of 1 Hz. In particular, the first subset includes 496,800 data records, which is equivalent to seven days of normal system operation without any unexpected interruption. Note that at the beginning of the operation (approximately 16,000 records) a transient period is present, and hence it is ignored in the analysis due to system instabilities. For the four remaining days of recording, namely in the second subset, 449,919 data records are included, with 36 attack scenarios that represent 11.98% of the subset’s data. Regarding each subset’s allocation, the first subset is utilized for training and validation of the CNN model, while the second one is deployed for online evaluation and testing.

**Figure 8:**
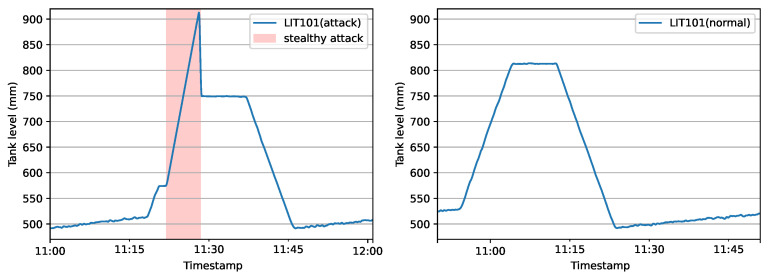
Stealthy attack (**left**) on the level sensor LIT101 in P1 of the SWaT testbed. Normal operation of a working cycle is illustrated in the **right** panel for comparison.

In general, most of the attacks are launched on the communication network before and after reaching the PLC, and specifically, on the sensor values and the actuator commands. Depending on the process stage and the number of attacked elements of the testbed, attacks are classified as follows: 26 Single-Stage–Single-Point attacks (SSSP); 4 Single-Stage–Multi-Point (SSMP) attacks; 2 Multiple-Stage–Single-Point (MSSP) attacks, and 4 Multiple-Stage–Multiple-Point (MSMP) attacks. Within the above-mentioned set of attacks, stealthy ones are also included, in which sensor values may be compromised in such a convincing way that the existing monitoring system is not able to detect them. Figure 8 illustrates a stealthy attack on the tank-level sensor from P1 (LIT101), while the right plot shows the temporal evolution of a normal filling/emptying cycle from the same tank. In particular, the red-shaded region signifies the limits of the launched attack in a stealthy manner, since as it is displayed, a slow increase of the water level takes place that finally leads to underflow of the tank.

### 4.2. Construction of Structural Causal Models

To build the structural causal models for detecting attacks in single actuators, an actuator–sensor (cause–effect) selection step is initially performed. We selected as the set of critical actuators the primary (P101) and secondary (P102) raw water pumps from the first stage (P1) and the chemical dosing pumps P203 and P204 from the second stage (P2) for the following reasons. First, any attack that is injected in the pumps from P1 will undoubtedly impact all downstream stages with irreversible consequences. Second, P2 is principally responsible for the overall water quality as it performs chemical dosing in case the measured values of the chemical compound are not within the prescribed quality levels. Any compromising of the chemical dosing station may have a devastating impact on the public health and must, in any case, be detected correctly and in a timely manner. Based on the causal analogy we introduced in the first section, we select the sensor parameters as the effect variables for each model. The level measuring parameters LI101 and LIT301 represent the effect variables $X_{s}^{1}$ and $X_{s}^{2}$ for the first and second model, respectively. Similar, the actuators P101, P102 and P203, P205 represent the cause variables $X_{a}^{1}$, $X_{a}^{2}$ for the first model and $X_{a}^{3}$, $X_{a}^{4}$ for the second model, respectively. Hence, we obtain two multivariate forecasting models to approximate the effect estimates:

(9) $${\hat{X}}_{s,t}^{1}\;=\;F_{1}\left(X_{a,(t-l:t-1)}^{2},X_{a,(t-l:t-1)}^{1}\right),X_{s,(t-l:t-1)}^{1}))$$

(10) $${\hat{X}}_{s,t}^{2}=F_{2}\left(X_{a,(t-l:t-1)}^{3}\right),X_{a,(t-l:t-1)}^{4}),X_{s,(t-l:t-1)}^{2})),$$

where *l* is the window length and $F_{1}$, $F_{2}$ are the non-linear functions estimated by the multivariate CNN models in P1 and P2, respectively. Due to high autocorrelation, the past of the effect variables $X_{s}^{1}$, $X_{s}^{2}$ is included in the input variables set.

On a univariate level, the focus is to detect any attacks on individual sensors by modeling their behavior under normal operating conditions. The proposed CNN architecture with causal dilations is employed for univariate autoregression of the investigated transmitted signals within the SWaT testbed. Table 2 summarizes the sensors that mainly contributed to the final attack detection. Nevertheless, we initially chose all continuous variables from the sensors to build the univariate models, as in practice all components in an ICS are candidates to be compromised by external attackers.

### 4.3. Detection Performance Metrics

**Figure 9:**
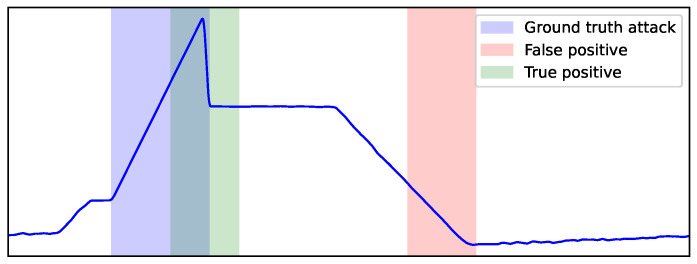
Example of true and false positive windows relative to the ground truth of an attack interval. An overlapping with the true attack interval (blue) yields a true positive window (green), while a non-overlapping window (red) yields a false positive one.

In the final evaluation, test records are classified either as “attack” or “normal”. Note that it is not necessary to explicitly define a specific threshold value for the anomaly detection, since ESD will either reject or accept the outlier hypothesis of the examined window in which causal effect from the prediction errors is estimated. Figure 9 illustrates an example of two segments that represent two “attack” predictions, with the ground-truth attack segment in blue. As we adopt a point-adjust scheme (or event-based), such as in [46,55], the green segment that overlaps the ground-truth attack is a *true positive* (*tp*), while the red segment that does not overlap the ground truth is treated as a *false positive* (*fp*). Lastly, *false negatives* (*fn*) represent the number of attacks that were missed.

Principally, any attack detection system aims to achieve a high detection rate with the lowest number of false alarms (e.g., one in a six month period [5]). Therefore, as a general measure of performance of the proposed approach, we use the F1 score (Equation (13)), which represents the harmonic mean of precision and recall. Precision (Equation (11)) takes into account the number of detected attacks that are not actual attacks and is formally expressed by the proportion of correctly predicted attacks of all the segments that are classified as positive. On the other hand, recall (Equation (12)) takes into account the number of true attacks that have been missed and is formally expressed by the proportion of the correctly inferred attack segments to the total number of events when the ICS is under attack.

(11) $$precision\;=\;\frac{tp}{tp\;+\;fp}$$

(12) $$recall\;=\;\frac{tp}{tp\;+\;fn}$$

(13) $$F1\;=\;2\;\times \frac{precision\;\times \;recall}{precision\;+\;recall}$$

### 4.4. Results

We first present all types of attacks (see Table 3) that were detected by the proposed approach across different process stages in the testbed, as well as the the model with the sensory signals that contributed to the detection. Multivariate predictions are primarily obtained by the predefined structural causal models for detecting individual attacks on actuators, as they model the dependencies with the signals acquired from level sensors LIT101 and LIT301. On the other hand, univariate models based on the sensor signals were able to capture attack patterns from both components in the system, namely sensors and actuators. This mainly happens due to the causal dependency between the actuator, which performs independent interventions (e.g., open/close valve), and the sensor via an underlying physical law. An example is Attack 17, in which the attack is launched on actuator MV303 that controls the water flow in the backwash process of the ultrafiltration unit of stage P3. Our approach detects the attack by utilizing the univariate model with the level signal from sensor LIT401 that measures the tank level in the reverse osmosis feed-water tank. This outcome seems quite natural, as any successful compromise of an actuator from P3 can be directly captured by a downstream component in stage P4.

To verify the performance of our method, we further analyze and compare its results from Table 3 with TABOR [44], a state-of-the-art technique for anomaly detection in ICSs that, besides timed automata, employs Bayesian networks to learn the causal structure and to estimate conditional probabilities. In some attacks, components are compromised at such a slow rate that they may be easily confused with normal behavior, and hence they are characterized as stealthy. Based on the evaluation in [44], none of the stealthy attacks (Attacks 3, 16, 41) in the level-sensory signals were detected by TABOR. Our causal-based approach, such as the CNN-based approach [14], not only detected these attacks via both univariate and multivariate models, but their sensory signals signified the component (level sensor) that was indeed under attack. The dual isolation-forest-based (DIF) framework, as is presented in [38], was capable of detecting stealthy Attacks 16 and 41, while Attack 3 was missed. Unfortunately, other state-of-the-art methods [46,55] did not report detailed listings of detected attacks for direct comparison.

**Figure 10:**
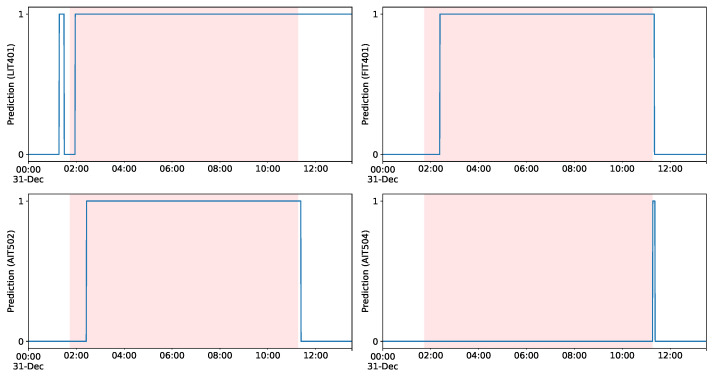
Detection of Attack 18 in SWaT testbed using four univariate models (LIT401, FIT401, AIT502, AIT504) with the proposed approach. Red region denote the ranges of the injected attack.

Within an ICS such as SWaT, it is naturally expected that strong interdependencies between various components are present, for which some of them may be correlational and others may be associated by causal relationships. Hence, it is of great importance to be able to flag the effects of an attack without having to incorporate the signal of the compromised component itself. An example of such a scenario is Attack 28, which is presented in Figure 10, via the predicted values of the corresponding univariate models. In particular, the shaded region shows the limits of Attack 28, in which the ultrafiltration feed-water pump is turned off by the attackers. Figure 10 shows that after a short period of time our detector predicts an anomalous state on sensory signals LIT401, FIT401, and AIT502. Interestingly, at the end of the attack, the univariate detector based on signal AIT504 (chemical analyzer) signifies an anomaly since the attack was successful, and the system takes immediate corrective measures by intervening with respect to the water’s chemical properties. After the attack, we notice that our model with level signal LIT401 correctly captures the recovery period, as it takes a while for the system to return to its initial normal state. Such a diagnostic scheme also enables experts to better evaluate the severity of and to ultimately understand the cascading effects of a launched attack in a CPS.

#### 4.4.1. Ablation Study

While achieving a high detection rate of cyber-attacks and their effects is essential for high performance, raising red flags as infrequently as possible, or even eliminating them, is equally important. We perform an ablation experiment for evaluating the impact of the causal effect estimation step from the proposed approach. To this end, no sliding window is performed on the predictions errors from the univariate models (AIT502, DPIT301, and FIT201), and hence, the test statistic from ESD is directly computed to obtain the predicted attacks. By comparing the results from Table 4, we observe that our approach performs better without raising any false alarm compared to using the aforementioned direct computation method. Specifically, the ESD method with sensory signal AIT502 exhibits a very high false alarm rate, as the predicted errors probably violate the normality assumption that it is required by the ESD test. These findings corroborate the denoising properties of the causal model we assumed due to removal of the confounding factors.

#### 4.4.2. Evaluation

A comprehensive comparison with the existing literature is summarized in Table 5 with respect to the most important evaluation metrics. Note that the results from the table are taken as reported from the considered papers, in which the SWaT dataset is utilized for evaluation per event. Two methods did not provide any exact number of the attacks, and therefore they are not listed. As shown in Table 5, our method outperforms all methods in the F1 score, and only the recent causality-based method [46] yields better results in recall. This highlights the power of incorporating causality for anomaly detection that may carry information about the causal mechanism.

The proposed approach successfully detects 32 out of 36 attacks, while the 1D CNN ensemble method [32] and TABOR [44] detect 31 and 24, respectively. According to [32], the method missed Attack 35, which was launched at stage P1 by shutting down both both water pumps P101 and P102. Such an attack scenario might cause a significant disruption in the system with many cascading effects. TABOR exhibits quite a low detection rate, which can be attributed to the fact that continuous values from sensory signals are discretized, and therefore, temporal patterns may be degraded. Even though the DIF method [38] detected most of the scenarios, it failed to detect Attack 22, which takes place at multiple stages and components in SWaT. The specific attack may trigger potential damage in the process of reverse osmosis. IDS-CNN [14] performed similarly to 1D CNN; however, the method did not recognize Attack 24, in which water quality in SWaT was compromised. Remarkably, our approach identifies all previous attacks that were missed by the alternative methods.

Interestingly, three methods [14,32,46], based on their F1 scores from Table 5, achieved high attack detection performance comparable with ours. These methods have in common the selection of 1D CNN architectures for the computation of the prediction errors. In addition, we apply the causal convolution so as to maintain and propagate across all layers the temporal ordering of the input time series. Previous findings confirm the effectiveness and ultimately the suitability of the proposed CNN architecture for anomaly detection purposes.

From Table 5, it is clear that our approach yields state-of-the-art performance, with an F1-score of 0.941, which is better than the method presented recently in [46]. However, we were not able to detect four attacks that were likewise missed by IDS-CNN [14]. In particular, Attacks 4, 13, 14, and 29 from SWaT did not reach their target, and hence the anomaly score from causal effect was negligible. For brevity, we refer the interested reader to the initial SWaT paper [54], which lists all details regarding the previously mentioned attack scenarios. In addition to the efficiency due to the compactness of the CNN architecture, the proposed approach achieves a zero false positive rate, which renders the method extremely useful for deployment in real-world ICSs.

## 5. Conclusions

In this work, we propose an unsupervised causality-based solution for detecting anomalies originated from cyber-attacks in a water treatment testbed. A novel anomaly score that is computed from the causal effect estimation ensures high robustness and accuracy. No threshold needs to be explicitly defined for the method to reach the final decision. Another distinguishing characteristic is the selection of the most important sensor signals from the univariate model approach, which yields a set of the root cause candidates. Based on our experiments on the SWaT testbed, we conclude that the prediction errors are successfully denoised in such a way that in the online phase no false alarms are triggered by the final detection mechanism, which is performed by the extreme stundentized deviate test. Our approach outperforms the existing state-of-the-art methods, thus verifying its utility for real-world applications. It is worth mentioning that the model for estimating the causal effect is limited by the assumption of the non-existence of latent confounding factors. In various cases, though, this assumption may not hold, which might affect the final detection result. Hence, more advanced techniques need to be applied to address this issue. Future research will focus on two major directions: first, building more complex causal graphs by enabling background domain knowledge to flow into causal-structure-learning algorithms; and second, applying our method to other cyber–physical systems, such as vehicle engine testbeds, which contain highly complex interdependencies. Last but not least, we will accordingly analyze the denoising performance of the causal anomaly score. 

## Figures and Tables

**Table 1:** Selected hyperparameters for univariate/multivariate 1D CNN models.

Parameters	Values
Sequence length (*l*)	20
No. of convolutional blocks	2
Size or average pooling	2
Filter size	3
No. of filters in the 1st convolutional block	(8, 16)
No. of filters in the 2nd convolutional block	(16, 32)
Dilation rates in the 1st convolutional block	(1, 2)
Dilation rates in the 2nd convolutional block	(4, 8)
No. of neurons in the fully connected layer (univariate)	16
No. of neurons in the fully connected layer (multivariate)	32

**Table 2:** Signals from sensors that contributed to the final attack detection.

Sensor Signals	Process Stage
LIT101, FIT101	P1
FIT201, AIT202	P2
DPIT301, LIT301, FIT301	P3
FIT401, LIT401	P4
AIT502, AIT504	P5

**Table 3:** Overview of all 32 detected attack scenarios injected in the SWaT testbed [54]. Sensor type in parenthesis denotes the corresponding CNN-based models.

Attack No.	Description	Model
1	Open MV101	univariate (LIT101), multivariate (LIT101)
2	Turn on P102	univariate (FIT201), multivariate (LIT101)
3	Increase water level in LIT101 by 1 mm every second (stealthy)	univariate (LIT101), multivariate (LIT101)
6	Set value of AIT202 to 6	univariate (AIT202), multivariate (LIT301)
7	Water level in LIT301 increased above HH	univariate (LIT301), multivariate (LIT301)
8	Set value of DPIT301 as >40 kPa	univariate (DPIT301, LIT401)
10	Set value of FIT401 as <0.7	univariate (FIT401)
11	Set value of FIT401 as 0	univariate (FIT401)
16	Decrease water level in LIT301 by 1 mm each second (stealthy)	univariate (LIT301), multivariate (LIT301)
17	Do not let MV303 open	univariate (LIT401)
19	Set value of AIT504 to 16 uS/cm	univariate (AIT504)
20	Set value of AIT504 to 255 uS/cm	univariate (AIT504)
21	Keep MV101 on continuously; Value of LIT101 set to 700 mm	univariate (LIT101), multivariate (LIT101)
22	Stop UV401; Value of AIT502 set to 150; Force P501 to remain on	univariate (FIT401, AIT502, AIT504)
23	Value of DPIT301 set to >0.4 bar; Keep MV302 open and P602 closed	univariate (DPIT301, LIT401)
24	Turn of P203 and P205	multivariate (LIT301)
25	Set value of LIT401 as 1000; P402 is kept on	univariate (LIT401)
26	P-101 is turned on continuously; Set value of LIT-301 as 801 mm	univariate (LIT301), multivariate (LIT101)
27	Keep P302 on continuously; Value of LIT401 set as 600 mm Set value of LIT301 as 801 mm	univariate (LIT401)
28	Close P302	univariate (LIT401, FIT401, AIT502, AIT504)
30	Turn P101 and MV101 on continuously; Set value of LIT-101 as 700 mm; P-102 started itself because LIT301 level became low	univariate (LIT101), multivariate (LIT101)
31	Set LIT401 to less than L	univariate (LIT401)
32	Set LIT301 to above HH	univariate (LIT301), multivariate (LIT301)
33	Set LIT101 to above H	univariate (LIT101), multivariate (LIT101)
34	Turn P101 off	multivariate (LIT101, LIT301)
35	Turn P101 off; Keep P102 off	multivariate (LIT101, LIT301)
36	Set LIT101 to less than LL	univariate (LIT101), multivariate (LIT101)
37	Close P501; Set value of FIT502 to 1.29	univariate (AIT502, AIT504)
38	Set value of AIT402 as 260; Set value of AIT502 to 260	univariate (AIT502)
39	Set value of FIT-401 as 0.5; Set value of AIT-502 as 140 mV	univariate (FIT401, AIT502)
40	Set value of FIT-401 as 0	univariate (FIT401, AIT502)
41	Decrease LIT301 value by 0.5 mm/s (stealthy)	univariate (LIT301), multivariate (LIT301)

**Table 4:** False positive rates with and without causal effect estimation.

Univariate Model	FPR w/o Causal Effect (%)	FPR with Causal Effect (%)
AIT502	10.8	0
DPIT301	1.2	0
FIT201	1.0	0

**Table 5:** Comparison of state-of-the-art attack detection techniques per event. NA signifies unavailability of the specific metric in the corresponding paper. Bold values denote the best performance in the correspnding metric.

Method	Precision	Recall	F1	No. of Detected Attacks
1D CNN ensemble method [32]	0.912	0.861	0.886	31
TABOR [44]	0.862	0.788	0.823	24
DIF [38]	0.935	0.835	0.882	30
USAD [55]	0.987	0.740	0.846	NA
IDS-CNN [14]	0.947	0.833	0.886	30
Causality-based AD [46]	0.945	**0.892**	0.918	NA
Our approach	**1.0**	0.889	**0.941**	**32**

## Data Availability

Publicly available datasets were analyzed in this study. These data can be found here: https://itrust.sutd.edu.sg/itrust-labs_datasets (accessed on 5 January 2021).
